# Epidemiological trends in the presentation of diabetic ketoacidosis in children newly diagnosed with type 1 diabetes from 2011 to 2017 in Kuwait

**DOI:** 10.3389/fendo.2022.908458

**Published:** 2022-12-09

**Authors:** Dalia Al-Abdulrazzaq, Fouzeyah Othman, Sarah Qabazard, Abeer Al-Tararwa, Dina Ahmad, Hala Al-Sanae, Hessa Al-Kandari

**Affiliations:** ^1^ Department of Pediatrics, Faculty of Medicine, Kuwait University, Kuwai City, Kuwait; ^2^ Department of Population Health, Dasman Diabetes Institute, Kuwai City, Kuwait; ^3^ Department of Pediatrics, Al-Farwaniyah Hospital, Ministry of Health, Kuwai City, Kuwait; ^4^ Department of Pediatrics, Al-Amiri Hospital, Ministry of Health, Kuwai City, Kuwait

**Keywords:** childhood, diabetic ketoacidosis, type 1 diabetes, type 1 – child – adolescent, Kuwait

## Abstract

**Background:**

Monitoring the trends in the presentation of T1D over decades cannot be underestimated as it provides a rich source of information on diabetes-related complications like DKA. DKA represents a medical emergency, with potentially fatal outcome, and thus the prevention of DKA is a priority in diabetes care. The aim of this study is to report on trends in the presentation of DKA in children newly diagnosed with T1D in Kuwait.

**Material and methods:**

This study is based on a retrospective review of children newly diagnosed with T1D aged 14 years or less at three Governmental Hospitals representing three health sectors out of the total six health sectors in the country during the period 2011-2017.

**Results:**

A total of 799 children (376 males and 423 females) were newly diagnosed with T1D. 287 children presented with DKA (35.9%) with only 73 children (9.1%) classified as severe. During the years 2011 to 2017, we note that the percentage of children older than 6 years of age presenting with severe DKA has decreased significantly (p=0.022). Unfortunately, this has not been replicated in children younger than 6 years.

**Conclusion:**

This study highlights the importance of continued monitoring of clinical characteristics of children at diagnosis of T1D specifically presenting with DKA to enable diabetes care professionals to appreciate the multifaceted aspects of T1D, in particular the importance of raising awareness of the early signs of the onset of T1D with special attention to DKA and its severe consequences.

## Background

The incidence of Type 1 Diabetes (T1D) in children has been increasing in the past few decades worldwide (1, 2). The situation in Kuwait is no exception, demonstrated by the fact that the incidence of T1D doubled since the 1990s resulting in an incidence rate of 40.9 per 100,000 children per year (3).

Monitoring the trends of diabetes in the country especially T1D as well as related complications, especially diabetic ketoacidosis (DKA) is crucial. Furthermore, monitoring the trends in the presentation of T1D over decades cannot be underestimated as it provides a rich source of information on diabetes-related complications like DKA. DKA is of a particular concern in this context as it is an acute complication of diabetes related to delays in the diagnosis of T1D and the general lack of awareness of the disease and its effects on health and wellbeing. In Kuwait, during the 90´s a large number (49.0%) of children newly diagnosed with T1D presented with DKA (4). DKA represents a medical emergency, with potentially fatal outcome, and thus the prevention of DKA is a priority in diabetes care. Special attention is clearly given to DKA in a child newly diagnosed with T1D as it is linked to higher risk of morbidity and mortality as well as associated with increased healthcare expenditure (5).

Multiple key factors had been suggested to play a role in the presentation of children newly diagnosed with T1D especially DKA. For example, awareness campaigns for both the public and health professionals have been found to be effective in raising the awareness of initial symptoms of T1D and thus encourage an early diagnosis (6). Another factor is socioeconomic status, socioeconomic inequalities, measured as low education and occupational levels, were associated with an increased probability of DKA at T1D diagnosis (7). Moreover, younger children have been reported to have a higher probability of DKA at diagnosis (7–11).

The aim of this study is to report on trends in the presentation of DKA in children newly diagnosed with T1D in Kuwait from 2011 to 2017, focusing primarily on children 6 years and older compared to younger children.

## Materials and methods

This study is based on a retrospective review of medical records of 799 children newly diagnosed with T1D aged 14 years or less at three secondary Governmental Hospitals during the period 2011-2017. Children newly diagnosed with diabetes in Kuwait are managed in diabetes centers at six secondary Governmental Hospitals representing a total of six health sectors in the country. However, three out of the six diabetes centers (799 (49.1%) out of 1630 children) were included in the study due to completion of the data at diagnosis. Children younger than the age of 6 months were excluded from the study due to the possibility of the diagnosis of neonatal diabetes in this age group.

Data were extracted using standard data forms. The forms included baseline information at the time of diagnosis such as demographic information, anthropometric measures (weight, height, and body mass index [BMI]) expressed as standard deviation scores (SDS) according to the World Health Organization (WHO) child growth standards which are adapted by the Ministry of Health (MOH) of Kuwait (12). BMI was categorized as underweight, normal, overweight, and obese based on the WHO growth standards. Measures of Hemoglobin A1C (HbA1C) was also collected. Children were categorized according to age older and younger than 6 years (6 years is the official age of enrolment to school in Kuwait). The 2018 International Society of Pediatric and Adolescent diabetes (ISPAD) guidelines (13) are approved by the Pediatric Council of the MOH in Kuwait and were used to confirm the diagnosis of T1D in these children, i.e. the presence of one or more pancreatic autoimmune antibodies (Anti-glutamic acid decarboxylase (GAD), anti-islet cell (ICA), anti-insulin (IAA), anti-protein tyrosine phosphatase (IA-2A) antibodies). DKA was defined as venous pH <7.3 or serum bicarbonate <15 mmol/L and further categorized as mild (venous pH <7.3, serum bicarbonate <15 mmol/L), moderate (pH <7.2, serum bicarbonate <10 mmol/L) or severe (pH <7.1, serum bicarbonate <5 mmol/L) as per the approved ISPAD guidelines (14).

Statistical analyses were performed using STATA software13.1. Differences with p-value of less than 0.05 were deemed to be statistically significant. Continuous variables were expressed as mean (SD) when normally distributed or median (interquartile range: IQR) otherwise. T-test or Kruskal–Wallis test were used to test for the differences in continuous variables as appropriate while Pearson’s chi-squared test and Fisher exact test were used to test for differences in categorical variables. Multiple logistic regression (Backward stepwise regression approach) was used to investigate the baseline factors associated with the presence of severe DKA in children older than 6 years of age.

The study was approved by the Ethics Review Committees at DDI (RA 2011-006 & RA 2015-010) and MOH (Ref 2017/651). The study was performed in accordance with the Declaration of Helsinki.

## Results

During the study period, a total of 799 children aged 14 years or less (376 males and 423 females, p=ns) were newly diagnosed with T1D and included in the study. 549 (68.7%) of children were 6 years or older at the time of diagnosis of T1D. Clinical characteristics of these patients at the time of diagnosis are shown in **Table 1**. Most of the children diagnosed with T1D (total n=514, 64.3%, <6 years n=143, 57.2%, and >6years n=371, 67.6%) were Kuwaiti nationals (p=0.005) while the rest represented other nationalities residing in Kuwait. No statistically significant differences between Kuwaiti and non-Kuwaiti children were observed in any of the characteristics evaluated and therefore, the entire group is presented as one data set.

**Table 1:** Baseline characteristics of children older than 6 years at diagnosis of T1D compared to younger children during the period 2011-2017.

Variable	Total (n=799, 100%)	Children < 6 years (n=250, 31.3%)	Children > 6 years (n=549, 68.7%)	P value
Sex, Male n (%)	376 (47.1%)	123 (49.2%)	253 (46.1%)	0.413
Median age in years, (IQR)	7.8 (5.1, 10.0)	3.7 (2.5, 5.0)	9.2 (7.6, 10.5)	N. A
Nationality, n (%)				0.005
Kuwaiti	514 (64.3%)	143 (57.2%)	371 (67.6%)	
Non-Kuwaiti	285 (35.7%)	107 (42.8%)	178 (32.4%)	
Mean weight Z score (SDS)	+0.30 (1.59)	+0.30 (1.59)	+0.49(1.65)	0.174
Mean height Z score (SDS)	+0.67 (2.14)	+0.67(2.14)	+0.29 (1.42)	0.005
Mean BMI Z score (SD)	-0.04 (1.93)	-0.04 (1.93)	0.28 (1.87)	0.031
BMI categories (n, %)				<0.001
Underweight	77 (10.4%)	22 (9.7%)	55 (10.6%)	
Normal	453 (61.0%)	174 (77.0%)	279 (54.0%)	
Overweight	100 (13.5%)	17 (7.5%)	83 (16.1%)	
Obese	113 (15.2%)	13 (5.8%)	100 (19.3%)	
Presence of DKA, n (%)	287 (35.9%)	96 (38.4%)	191 (34.8%)	0.324
Severe DKA, n (%)	73 (9.1%)	22 (8.1%)	51 (8.5%)	0.487
Mean HbA1C % (SD)	11.4 (2.3)	10.4 (2.1)	11.8 (2.3)	<0.001

T1D, Type 1 Diabetes; IQR, Interquartile range; BMI, body mass index; DKA, diabetes ketoacidosis; HCO, bicarbonate; HbA1c, hemoglobin A1c.

Out of a total 799 patients, 287 children presented with DKA (35.9%) with only 73 children (9.1%) classified as severe. There was no significant difference between children younger than 6 years of age with regards to the presence of DKA or severe DKA at the time of diagnosis compared to older children. As expected, younger children had a lower mean HbA1C at presentation (10.4% ± 2.1 vs 11.8% ± 2.3, p<0.001).

In **Tables 2** and **3** we describe baseline characteristics of children presenting with DKA compared to those who did not present with DKA, and severe DKA compared to those who presented with non-severe DKA (mild or moderate) at the time of diagnosis of T1D respectively. As expected, children who presented with DKA were leaner (BMI Z score -0.13 ± 2.11 vs +0.35 ± 1.74, p<0.001) and had higher HbA1C (12.0 ± 2.2 vs 11.0 ± 2.3, p<0.001) at diagnosis compared to those who did not present with DKA (**Table 2**). There was no significant difference between children presenting with severe DKA with regards to gender, age, nationality, and growth parameters (weight, height, and BMI) compared to children who presented with non-severe DKA at the diagnosis of T1D (**Table 3**). Although statistically not significant, children presenting with severe DKA had predictably slightly higher HbA1c (12.4 ± 2.2 vs 11.8 ± 2.2, p=0.051) (**Table 3**).

**Table 2:** Characteristics of children presenting with DKA compared those who did not present with DKA at diagnosis with T1D in Kuwait.

	DKA (n=287, 35.9%)	No DKA (n=512, 64.1%)	P value
Sex, Male n (%)	128 (44.6%)	248 (48.4%)	0.297
Median age in years, (IQR)	7.9 (4.3, 9.9)	7.8 (5.1, 10.0)	0.324
Nationality, n (%)			0.24
Kuwaiti	177 (61.7%)	337 (65.8%)	
Non-Kuwaiti	110 (38.3%)	175 (34.2%)	
Mean weight Z score (SDS)	+0.16 (1.63)	+ 0.55 (1.61)	<0.001
Mean height Z score (SDS)	+0.42 (1.90)	+0.40 (1.55)	0.833
Mean BMI Z score (SD)	-0.13 (2.11)	+0.35 (1.74)	<0.001
BMI categories (n, %)			0.045
Underweight	37 (14.1%)	40 (8.3%)	
Normal	161 (61.4%)	292 (60.7%)	
Overweight	30 (11.5%)	70 (14.5%)	
Obese	34 (13.0%)	79 (16.5%)	
Mean HbA1C % (SD)	12.0 (2.2)	11.0 (2.3)	<0.001

T1D, Type 1 Diabetes; IQR, Interquartile range; BMI, body mass index; DKA, diabetes ketoacidosis; HCO, bicarbonate; HbA1c, hemoglobin A1c.

**Table 3:** Characteristics of children presenting with severe DKA vs non-severe DKA at diagnosis with T1D in Kuwait.

	Total (n=287, 100.0%)	Severe DKA (n=73, 25.4%)	Non-severe DKA (n=214, 74.6%)	P value
Sex, Male n (%)	128 (44.6%)	31 (42.5%)	97 (45.3%)	0.671
Median age in years, (IQR)	7.9 (4.3, 9.9)	8.3 (5.2, 9.9)	7.7 (4.1, 9.8)	0.523
Nationality, n (%)				0.262
Kuwaiti	177 (61.7%)	41 (23.2%)	136 (76.8%)	
Non-Kuwaiti	110 (38.3%)	32 (29.1%)	78 (70.9%)	
Mean weight Z score (SDS)	+0.16 (1.6)	-0.11 (1.8)	+0.25 (1.6)	0.165
Mean height Z score (SDS)	+0.42 (1.9)	+0.41 (1.92)	+0.43 (1.9)	0.964
Mean BMI Z score (SD)	-0.13 (2.1)	-0.45 (1.7)	-0.03 (2.2)	0.165
BMI categories (n, %)				0.225
Underweight	37 (14.1%)	12 (18.4%)	25 (12.7%)	
Normal	161 (61.5%)	38 (58.5%)	123 (62.4%)	
Overweight	30 (11.4%)	10 (15.4%)	20 (10.2%)	
Obese	34 (13.0%)	5 (7.7%)	29 (14.7%)	
Mean HbA1C % (SD)	11.9 (2.2)	12.4 (2.2)	11.8 (2.2)	0.051

T1D, Type 1 Diabetes; IQR, Interquartile range; BMI, body mass index; DKA, diabetes ketoacidosis; HCO, bicarbonate; HbA1c, hemoglobin A1c.

**Figure 1:**
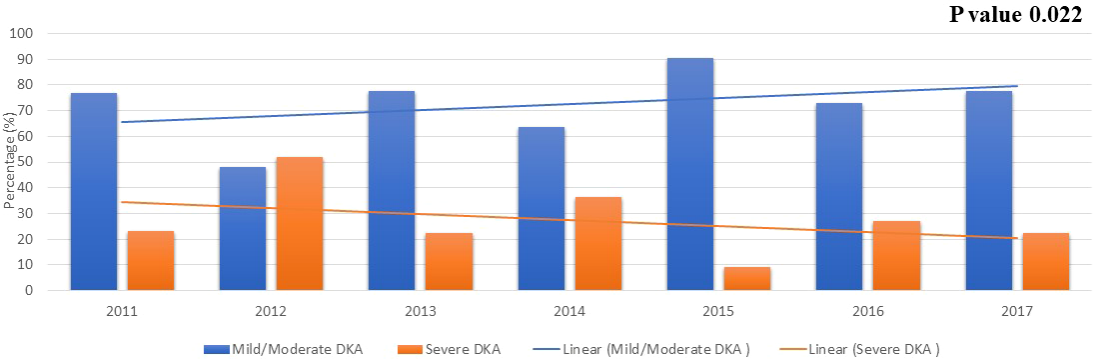
The severity of DKA over the years in children newly diagnosed with T1D older than 6 years of age. .

During the period from 2011 to 2017, we note that the percentage of older children presenting with severe DKA has decreased significantly (**Figure 1**, p=0.022). Unfortunately, this has not been replicated in children younger than 6 years. No trends were observed in anthropometric measures and HbA1C at diagnosis during the study period.

**Table 4** shows the association between baseline characteristics and the presentation of severe DKA at diagnosis in children older than 6 years of age with T1D using logistic regression. None of the baseline characteristics showed a significant association with DKA severity at the time of diagnosis.

**Table 4:** Predictive factors, using multiple logistic regression analysis, of presenting with severe DKA in patients older than 6 years of age at diagnosis of T1D.

Variables at diagnosis	OR	95% CI	*P* value
Male sex	1.04	0.37 - 3.00	0.356
Nationality	0.61	0.21 - 1.76	0.692
HbA1C	1.05	0.82 - 1.33	0.706
Weight SDS	0.72	0.13 - 3.98	0.704
Height SDS	1.21	0.45 - 3.29	0.707
BMI SDS	1.19	0.43 - 3.25	0.74

T1D, Type 1 Diabetes; OR, Odds ratio; CI, Confidence interval; BMI, body mass index; DKA, diabetes ketoacidosis; HbA1c, hemoglobin A1c.

## Discussion

This study describes the trends in presentation of DKA in children newly clinical characteristics diagnosed with T1D during the period from 2011 to 2017. The study revealed that 35.9% of children present with DKA at the time of diagnosis and only 9.1% were classified as severe. Most importantly, our data shows a decrease in presentation with severe DKA in children older than 6 years of age during the study period which has not been replicated in younger children.

Our study reports that about one third of all children diagnosed with T1D presented with DKA (35.9%). Although a high proportion of DKA among newly diagnosed children was found, these data demonstrate a significant reduction compared to previous reports during the 1990´s when nearly half (49.0%) of all children with newly diagnosed T1D presented with DKA (4). However, over many years, the DKA prevalence among newly diagnosed children with T1D in Kuwait has remained at a fairly constant rate with 37.7% between 2000-2006 (15) and 33.6% between 2011-2013 (16). International reports demonstrate large variation in the rate of DKA at the diagnosis of T1D in children and, for example, our study reports on a higher rate compared to Sweden (19.5%), Denmark (20.7%), Norway (22.1%) and the German-Austrian registry of Diabetes (Diabetes-Patienten-Verlaufsdokumentation, DVP) (19.8%) but lower compared to reports from Malaysia (66.7%) and Luxembourg (43.8%) (8, 17, 18). However, the rate of DKA in Kuwait is comparable to the data reported from the U.S. based on the Diabetes Pediatric Consortium (34.0%), the SEARCH study (36.9%), Saudi Arabia (37.7%), and Italy (38.5%) (5, 8, 19, 20). Further reports from the SEARCH study published recently showed increase in the DKA prevalence from 35.3% in 2010 to 40.6% in 2016 opposite to our DKA rates from 1990s till the study period (21).

In our study, children presenting with DKA were leaner i.e., had lower BMI z-scores, compared to children who did not. There have been similar reports with regards to BMI association with DKA at diagnosis of T1D where lower BMI was associated with higher rates of DKA (10, 22). This might be explained by lower β-cell residual function at the onset of T1D in lean children compared to obese and overweight children (23). In this study, we also report that children presenting with DKA at diagnosis had higher HbA1C which might reflect delay in the diagnosis of diabetes (9, 14, 24). This has been replicated in other international reports where higher HbA1C was associated with higher DKA in children with T1D (25).

During the study period, 9.1% of children newly diagnosed with T1D in the country presented with severe DKA. This rate has not changed compared to report published by Shaltout et al, at 8.8% (16). However, this rate is lower compared to neighboring countries in the region like Saudi Arabia which reported a 12.3% rate of severe DKA at diagnosis with T1D between 2005-2015 (5). However, similar to our study, no difference was found with regards to occurrence of severe DKA in children older than 6 years of age compared to younger children (5). The DPV registry had reported recently a slightly lower rate of severe DKA in children newly diagnosed with T1D between the period of 200-2019 (6.1%) (18). Different from our study, the DPV registry reports that children less than 6 years of age were more likely to present with severe DKA (18).

It is therefore of great concern that the rate of DKA in general and severe DKA in all age groups in Kuwait had remained at a relatively constant level with no evidence of improvements since the early 2000´s despite the presence of readily available access to medical care across the country. In this context, it should be mentioned that children newly diagnosed with diabetes in the country are referred and managed to diabetes centers at six secondary Governmental Hospitals with readily available access to medical care for both Kuwaiti-nationals and non-nationals. Nevertheless, it was very encouraging to find a significant decrease in in the overall trend in the presentation of severe DKA among children older than 6 years during 2011 to 2017 (**Figure 1**). However, there has been fluctuations in the rate of severe DKA during the study period in this age group, specifically the year 2012 with the highest rate of severe DKA during the study period (**Figure 1**) Although, during the study period, there has been no changes in access to medical services in the country nor change in diagnosis and management guidelines for diabetes in children. However, the overall trend in severe DKA presentation in children older than 6 years of age during the study period showed a decrease and the specific change in the year 2012 might reflect normal variation/fluctuation rather than specific causation. Risk factors namely, younger age, delayed diagnosis, ethnic minority, lower socioeconomic status, and residing in countries with low incidence/prevalence of T1D have been associated with DKA and probably its severity (14, 26). It should be noted that Kuwait is a country with one of the highest incidences of T1D in the world with a recent incidence of 41.7 per 100,000 per year (3). In our study, there was no significant difference between children presenting with severe DKA and those with non-severe DKA with regards to age and nationality (**Table 2**). However, children presenting with severe DKA had a slightly higher HbA1C at diagnosis which might reflect delay in seeking medical care resulting in delayed diagnosis. Furthermore, in children older than 6 years of age, there was no significant effect of any of the studied confounders on the presentation with severe DKA (**Table 3**). Therefore, we are inclined to assume that such improvements in the rates of severe DKA across the follow-up period in older children might be because of the establishment of the Childhood-Onset Diabetes electronic Registry (CODeR) in Kuwait from 2011 (3). Similar to other registries, this registry serves as an important tool for clinicians as a basis for health care planning and for researchers focusing on the clinical aspects and epidemiology of T1D. Registries provide invaluable support to the design and implementation of health care planning, national diabetes management programs and public health initiatives. In addition, national registries can also play a crucial role in the development of community education programs to increase awareness of T1D in the general population. Since the implementation of the CODeR in Kuwait in 2011, a community awareness campaign has been launched educating the public about symptoms of diabetes in children. The support such registries and campaigns provide over time has been reported in similar studies (27–29). Although the support provided by the national registry is universal for health care providers and families of children of all age groups, but it is still a challenge to achieve a decrease in the rates of severe DKA in younger age groups. This is a vulnerable young population and require special attention as the signs and symptoms of diabetes are nonspecific and might explain the delay in their disease recognition (30). Furthermore, severe DKA in younger children has been attributed to infections (31), dehydration and a severer form of metabolic decompensation (32).

Finally, our study highlights the importance of a continuous diabetes monitoring to provide opportunities for longitudinal designs to evaluate trends in characteristics of patients at diagnosis with T1D in a country over several years. To compare data internationally, we adhered to the definition of DKA, based on standardized measures as per the 2018 ISPAD guidelines (13). However, the study period (2011–2017) was relatively short which limits the possibility to identifying trends within the population. Moreover, some data from centers participating in the CODeR were not included in the analysis due to the large amount of missing data registered. Also, a limitation to our study is that information on socio-economic factors, season of diagnosis and additional biochemical tests like c-peptide levels were not available and therefore we could not study some of the confounding factors that might influence characteristics of children newly diagnosed with T1D and presenting with DKA in the country.

In conclusion, this study highlights the importance of continued monitoring of clinical characteristics of children at diagnosis of T1D specifically presenting with DKA to enable diabetes care professionals to appreciate the multifaceted aspects of T1D, in particular the importance of raising awareness of the early signs of the onset of T1D with special attention to DKA and its severe consequences. These aspects need much more attention and should be considered as a priority when planning future diabetes care strategies.

## Data availability statement

The datasets presented in this article are not readily available because the data that support the findings of this study are available from the MOH and DDI but restrictions apply to the availability of these data, which were used under license for the current study, and so are not publicly available. Data are however available from the corresponding author upon reasonable request and with permission of the MOH and DDI. Requests to access the datasets should be directed to dalia.alabdulrazzaq@ku.edu.kw.

## Ethics statement

The studies involving human participants were reviewed and approved by the ethical committee at the Ministry of Health of Kuwait (Ref 2017/651) and the ethical committee of Dasman Diabetes Institute (RA 2011-006 & RA 2015-010). A waiver of consent was granted by the ethical committee of the Ministry of Health of Kuwait and the ethical committee of Dasman Diabetes Institute. Written informed consent from the participants’ legal guardian/next of kin was not required to participate in this study in accordance with the national legislation and the institutional requirements.

## Author contributions

All authors have contributed significantly to this manuscript and in keeping with the latest guidelines of the International Committee of Medical Journal Editors. Authors contributions were as follows: DA-A was principal investigator of this project, she was responsible for the conception, planning, data management and analysis and conducting the study. She had drafted this manuscript. FO, AA-T, DA, and HA-S were responsible for data collection and data management. SQ was responsible for formatting and reviewing the manuscript in preparation for submission. HA-K had participated in the planning, data management, and conducting the study. All co-authors had participated in the review of this manuscript. All authors contributed to the article and approved the submitted version.
